# Auditory training changes temporal lobe connectivity in ‘Wernicke’s aphasia’: a randomised trial

**DOI:** 10.1136/jnnp-2016-314621

**Published:** 2017-03-04

**Authors:** Zoe VJ Woodhead, Jennifer Crinion, Sundeep Teki, Will Penny, Cathy J Price, Alexander P Leff

**Affiliations:** 1 Department of Brain Repair and Rehabilitation, University College London, London, UK; 2 Wellcome Trust Centre for Neuroimaging, University College London, London, UK; 3 Department of Experimental Psychology, University of Oxford, Oxford, UK; 4 Institute of Cognitive Neuroscience, University College London, London, UK; 5 Department of Physiology, Anatomy and Genetics, University of Oxford, Oxford, UK

**Keywords:** Wernicke’s aphasia, speech comprehension, phonological training, pharmacological trial, magnetoencephalography

## Abstract

**Introduction:**

Aphasia is one of the most disabling sequelae after stroke, occurring in 25%–40% of stroke survivors. However, there remains a lack of good evidence for the efficacy or mechanisms of speech comprehension rehabilitation.

**Trial Design:**

This within-subjects trial tested two concurrent interventions in 20 patients with chronic aphasia with speech comprehension impairment following left hemisphere stroke: (1) phonological training using ‘Earobics’ software and (2) a pharmacological intervention using donepezil, an acetylcholinesterase inhibitor. Donepezil was tested in a double-blind, placebo-controlled, cross-over design using block randomisation with bias minimisation.

**Methods:**

The primary outcome measure was speech comprehension score on the comprehensive aphasia test. Magnetoencephalography (MEG) with an established index of auditory perception, the mismatch negativity response, tested whether the therapies altered effective connectivity at the lower (primary) or higher (secondary) level of the auditory network.

**Results:**

Phonological training improved speech comprehension abilities and was particularly effective for patients with severe deficits. No major adverse effects of donepezil were observed, but it had an unpredicted negative effect on speech comprehension. The MEG analysis demonstrated that phonological training increased synaptic gain in the left superior temporal gyrus (STG). Patients with more severe speech comprehension impairments also showed strengthening of bidirectional connections between the left and right STG.

**Conclusions:**

Phonological training resulted in a small but significant improvement in speech comprehension, whereas donepezil had a negative effect. The connectivity results indicated that training reshaped higher order phonological representations in the left STG and (in more severe patients) induced stronger interhemispheric transfer of information between higher levels of auditory cortex.

Clinical trial registration

This trial was registered with EudraCT (2005-004215-30, https://**eudract**.ema.europa.eu/) and ISRCTN (68939136, http://www.isrctn.com/).

## Introduction

Speech comprehension impairments after stroke caused by damage to dominant temporoparietal cortex (ie, ‘Wernicke’s aphasia’ (WA) and global aphasia)^1–3^ are resistant to treatment by conventional methods^4^; hence, there is a need for new evidence-based therapies to improve auditory comprehension in aphasia.

One popular target for WA therapy is the low-level deficit in auditory phonological analysis.^5–7^ This typically involves training auditory discrimination with phonemes, consonant-vowel-consonant segments or longer sequences. Previous attempts have met with mixed results: whereas two case studies reported positive effects on auditory discrimination and/or comprehension,^8 9^ a further case study^10^ and case series of eight patients^11^ failed to find any significant improvements. We tested the efficacy of phonological training using Earobics software^12^ in a larger sample (n=20).

We also tested whether donepezil, an acetylcholinesterase inhibitor, could improve behavioural outcomes. Studies in rats and bats have shown that modulating the cholinergic pathway from nucleus basalis to auditory cortex enhances experience-dependent plasticity,^13^ whereas suppression of the cholinergic system reduces it.^14 15^ As the cholinergic system is specifically involved in behaviourally dependent learning,^16^ cholinergic drugs may be most effective when paired with behavioural therapy. Two clinical trials in aphasia by Berthier and colleagues^17 18^ administered donepezil in conjunction with conventional speech and language therapy. The drug improved aphasia quotient scores, particularly on picture naming. While Berthier and colleagues studies included patients with aphasia of all types, we focused on Wernicke’s and global aphasia and tested whether aphasia severity interacted with the efficacy of both phonological and donepezil therapies using a within-subject, cross-over design.

As well as behavioural outcome measures, we employed functional neuroimaging to investigate the neural mechanisms of the therapeutic effect. Following Teki and colleagues,^19^ changes in the auditory cortex’s sensitivity to phonological contrasts were assayed using the mismatch negativity response (MMN) recorded with magnetoencephalography (MEG). Participants were habituated to a repeated spoken syllable (the standard stimulus). Occasionally, syllables that differed acoustically or phonologically from the standard stimulus were presented (the deviant stimuli). The surprise elicited by these unanticipated deviant stimuli results in a stronger evoked neural response, known as the MMN.^20^ By comparing MMN responses with acoustic and phonemic deviants, we could reveal when and where neurons were sensitive to the phonological content of the stimuli.

Dynamic causal modelling (DCM)^21–23^ was used to investigate how the MMN arises from interactions between multiple neuronal sources in the temporal lobes. Teki and colleagues showed that in controls the difference between acoustic and phonemic deviants was reflected in stronger local adaptation of representations in the bilateral primary auditory cortex (Heschl’s gyrus (HG)) and the auditory association cortex (superior temporal gyrus (STG)). Patients with aphasia showed no such adaptation in the left HG or STG, but instead showed stronger feedforward connectivity on the right hemisphere. This study involved the same participants, MMN paradigm and analysis, but employed a longitudinal design to test the impact of phonological training and donepezil on connectivity. We hypothesised that an effective therapy would increase updating of phonological representations on the left hemisphere and decrease reliance on the right hemisphere regions.

## Materials and methods

### Study design

Our objectives were to investigate (1) whether phonological training improved speech comprehension, (2) whether donepezil facilitated phonological training effects and (3) the impact of the therapies on directed connectivity within the auditory network.

**Figure 1:**
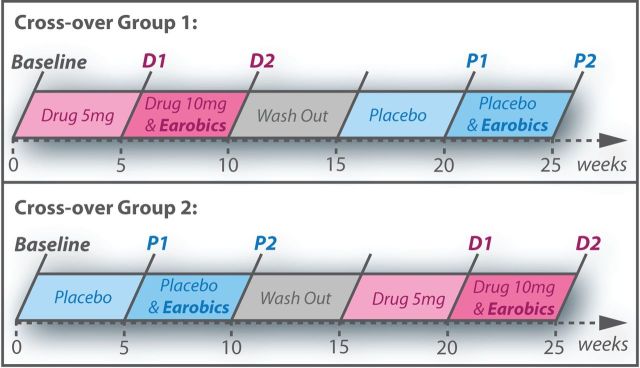
Study design showing order of the five time points (baseline, **D1, D2, P1 and P2**) for the two cross-over groups.

A randomised, double-blind, placebo-controlled cross-over design was used (figure 1). Each participant received four 5-week blocks of treatment:

- drug only (5 mg daily dose of donepezil)
- drug and Earobics (10 mg daily dose of donepezil, plus two 40 min daily sessions of Earobics)
- placebo only
- placebo and Earobics.

Participants were assessed at baseline, then before (1) and after (2) phonological training, in both drug (D) and placebo (P) conditions, leading to five distinct assessment time points: baseline, D1, D2, P1 and P2. This design allowed a factorial analysis of behavioural and effective connectivity outcome measures, comparing performance before (D1 and P1) versus after phonological training (D2 and P2), and when participants were receiving donepezil (D1 and D2) versus placebo (P1 and P2). There were no predetermined criteria for termination of the trial or the definition of outliers.

Participants were assigned to one of two cross-over groups (drug then placebo or placebo then drug). Block randomisation with bias minimisation was used: a computer algorithm generated a random number to allocate each new participant to a group. An independent researcher ensured that the cross-over groups did not become unbalanced by more than four patients. Participants, caregivers and researchers were blind to block allocation. Randomisation codes were held by the study pharmacist. Tablets were supplied in bottles labelled with the block number when they should be taken (1–4). Each bottle contained 35 indistinguishable lactose encapsulated tablets of placebo, 5 mg donepezil hydrochloride or 10 mg donepezil hydrochloride.

Outcome measures were prospectively selected. The primary behavioural outcome measure was score on the comprehensive aphasia test (CAT)^24^ speech comprehension scale (including word, sentence and paragraph comprehension; maximum score=66, aphasia cut-off=56), which was chosen for its high ecological validity. Other CAT measures (written comprehension, speech repetition, naming, reading and writing) and a sustained attention to response test (SART)^25^ were the secondary outcomes. The primary neuroimaging outcome was the effect of phonemic deviants on effective connectivity within the auditory network.

### Participants

**Figure 2:**
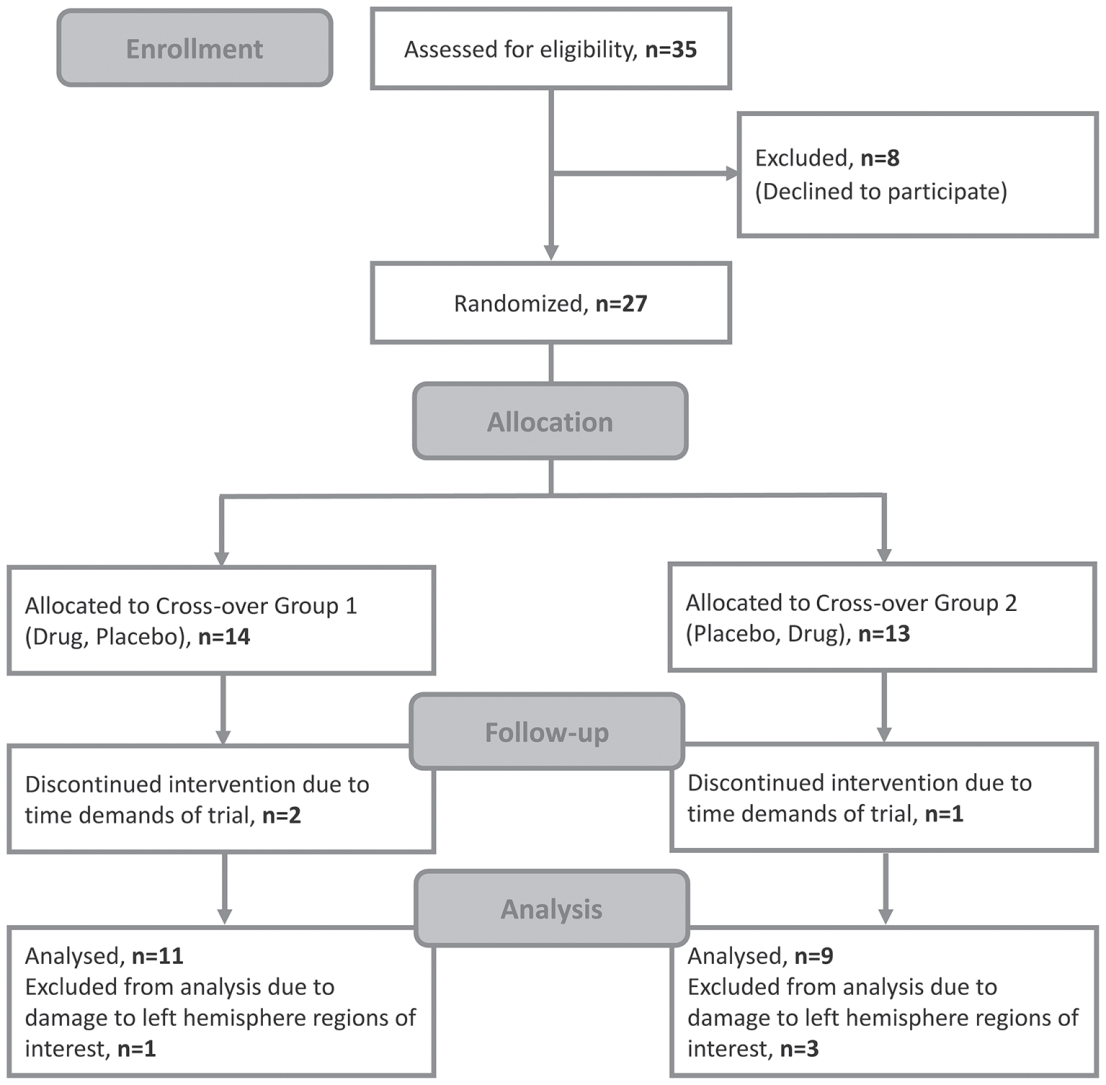
Consolidated Standards of Reporting Trials (CONSORT) trial flow diagram.

The participants were patients with chronic post-stroke aphasia. As the study required approval from the Medicines and Healthcare Products Regulatory Agency, which deemed the study a clinical trial of an investigational medicinal product, the power calculation was based on the drug effect. The best evidence available at the time of designing the study (from Berthier and colleagues, who used donepezil to treat post-stroke aphasia),^17^ indicated that 20 patients would be required to detect a 5% difference given a 7% within-person SD, 5% significance level and 90% power. Data collection occurred from November 2006 to August 2009 and ended when 20 patients had completed the protocol. Twenty-seven patients were enrolled, three withdrew after the baseline time point due to the trial’s time demands and a further four were excluded from the analysis as extensive left auditory cortex damage made them unsuitable for the DCM analysis (figure 2). We report the results from the remaining 20 patients. Baseline data from all 20 participants were previously reported by Teki and colleagues.^19^

All participants had left hemisphere stroke and normal hearing (three female; mean age (range)=62.4 (43–90) years; average time since stroke=3.3 (0.6–8.6) years; average lesion volume=127.3 (24.2–403.6) cm^3^). The demographics, lesion details and baseline behavioural scores are shown in table 1. The behavioural inclusion criteria were speech production impairment in CAT repetition and/or CAT naming, and speech comprehension impairment in CAT spoken word comprehension, CAT spoken sentence comprehension and/or a custom-made vowel identification task.

**Table 1:** Demographics, lesion details and baseline behavioural performance of the patients with aphasic stroke

ID	Group	Sex	Hand- edness	Severity	Aphasia subtype	Age at baseline (years)	Time since stroke (years)	Type of stroke	Lesion volume (cm^3^)	% of ROI damaged		Speech comprehension			Speech production	
										Left A1	Left STG	CAT word comp. (/30)	CAT sentence comp. (/32)	Vowel ID (/40)	CAT repetition (/74)	CAT object naming (/48)
1	1	M	L	M	G	69.6	1.0	I	69.5	1.8	0.0	29	28	**27**	**28**	**6**
2	2	M	R	M	W	62.7	1.2	I	42.4	7.2	21.1	30	**22**	38	**34**	**38**
3	1	M	R	M	W	63	8.6	I	429.3	81.0	43.3	27	**21**	**17**	**50**	**22**
4	1	M	L	M	W	61.5	7.6	I	314.0	52.9	0.0	**23**	**25**	**16**	**38**	**12**
5	1	M	R	M	W	67.8	7.4	H	64.3	8.1	30.5	29	**21**	37	**50**	**30**
6	2	M	R	M	W	60.5	5.6	I	161.1	52.7	9.3	27	30	**34**	**54**	**27**
7	1	M	R	M	W	64.9	1.2	I	171.1	39.7	17.9	27	**21**	38	**60**	**33**
8	1	M	R	S	G	61.4	1.9	I	242.9	52.6	52.6	**24**	**6**	**16**	**0**	**0**
9	2	F	R	S	G	66.5	5.3	I	195.2	42.3	21.4	**21**	**15**	**13**	**23**	**12**
10	1	M	R	M	W	61.5	3.4	M-L*	1.6	0.0	0.0	26	**26**	40	**63**	**36**
11	1	M	R	S	G	63.3	0.6	I	69.0	8.3	33.5	**20**	**13**	**12**	**0**	**0**
12	2	F	R	S	G	43.5	1.3	I	69.7	68.7	46.1	**14**	**17**	**17**	**18**	**14**
13	2	F	R	S	W	46.3	0.7	I	31.7	60.9	15.3	26	**12**	**26**	**26**	**41**
14	2	M	R	S	G	71.1	5.1	I	151.1	79.5	29.8	**20**	**14**	**7**	**0**	**0**
15	2	M	R	S	G	62.4	3.7	I	168.6	20.2	0.0	**22**	**11**	**21**	**30**	**0**
16	1	M	R	M	W	60.9	3.0	H	136.6	11.7	1.5	28	**26**	40	68	**37**
17	1^†^	M	R	M	G	45.4	3.7	I	61.2	5.8	16.7	27	**21**	39	**31**	**18**
18	2	M	R	M	W	74.7	0.6	I	41.1	7.3	2.9	**24**	**26**	**24**	**45**	**14**
19	2	M	R	M	G	50.2	1.8	I	280.5	29.5	0.9	**25**	**26**	39	**28**	**2**
20	1	M	R	M	W	90.3	3.7	I	62.9	14.2	0.0	27	**21**	**34**	68	**43**

Behavioural measures included CAT spoken word comprehension (score cut-off for impaired performance=25), CAT spoken sentence comprehension (cut-off=27), vowel identification (36), CAT total repetition score (67) and CAT object naming (43). Values highlighted in bold indicate scores below the threshold for normal performance.

*Patient 10 had four small lacunes in the left hemisphere: (1) superior and lateral occipital lobe (I); (2) deep to the superior frontal sulcus (I); (3) superior longitudinal fasciculus (L) and (4) inferomedial thalamus (L).

†Patient 17 failed to escalate to 10 mg of donepezil in the second drug block and remained on 5 mg for both blocks.

CAT, comprehensive aphasia test; I, infarct; H, haemorrhagic; M-L, multilacune; ROI, region of interest; STG, superior temporal gyrus.

During recruitment, it was assumed (wrongly as it turned out) that the participants’ scores on the main outcome measure would be normally distributed. It became apparent after the study closed that they fell into two groups (moderate and severe), as discussed in a previous report by Schofield and colleagues.^26^ None of the participants had the sample mean as their baseline score. A cluster analysis confirmed this bimodal distribution. We then treated the two groups as a factor in all further analyses, including when testing the main hypothesis. Group membership, moderate or severe, is listed in table 1.

The participants were classified into aphasia subtypes depending on their speech production abilities. Those whose object naming and repetition total scores placed them in the bottom quartile for subjects with aphasia in the Predicting Language Outcome and Recovery After Stroke (PLORAS) database^27^ were classed as global (G); otherwise we classified them as Wernicke’s aphasics (W). The majority of severe patients (six out of eight) had global aphasia (see table 1). A χ^2^ test showed no significant differences between the two therapy groups in terms of aphasia subtype (c^2^(1, n=20)=0.74, p=0.65).

All participants provided written informed consent according to the Declaration of Helsinki. Data were collected at the Institute of Neurology, University College London, and the study was approved by the Joint Research Ethics Committee of the National Hospital for Neurology and Neurosurgery and the Institute of Neurology, University College London.

### Phonological training

The phonological training software was Earobics version 1 for adolescents and adults.^12^ Earobics cycles through six independently adaptive tasks: (1) ‘memory matrix’, environmental sound-to-picture matching to train auditory short-term memory; (2) ‘sound check’, two-alternative forced-choice task to probe grapheme-to-phoneme mapping; (3) ‘get rhythm’, non-speech and speech sounds to probe auditory segmentation; (4) ‘connectivity’, compound word-to-picture matching to train phonological blending; (5) ‘rhyme time’, rhyme detection task using sequences of increasing numbers of words and (6) ‘same-different’, auditory discrimination using phoneme and word pairs. The participants were asked to complete 10 hours of training per week over each 5-week training block and record training duration in self-report diaries with some help from their partner or carer.

### Drug administration and monitoring

Donepezil dosage was determined according to the British National Formulary guidelines, starting at 5 mg for the first 5-week block. If this was tolerated, dose was escalated to 10 mg for the second block. All patients except one (patient 17) escalated to the higher dose. Adverse effects were monitored at every time point using a checklist of all possible adverse events (see online supplementary table).

10.1136/jnnp-2016-314621.supp1 Supplementary file

### Behavioural outcome measures

The CAT^24^ was administered at every time point as shown in figure 1. Only data from D1, D2, P1 and P2 time points were entered into the statistical analysis. Domain-general (non-verbal) effects of donepezil were tested using a modified version of the SART, a Go/No-Go task loading on sustained attention,^25^ presented with E-Prime^28^ and administered at baseline, D2 and P2 sessions only. The SART outcome measures included average reaction time for correct Go trials, accuracy of response inhibition for No-Go trials and post-error slowing. We also collected carer-reported measures of activity and participation from the Social Communication and Daily Planning from the Functional Assessment of Communication Skills for Adults (ASHA FACS)^29^ at all time points.

### Structural MRI

A T1-weighted structural brain image with 1 mm^3^ voxel size was acquired with a Siemens Sonata 1.5T MRI scanner at baseline. The Automated Lesion Identification toolbox^30^ for SPM8^31^ identified lesion location and volume.

### MEG and electroencephalogram data acquisition

The evoked response potentials were collected at D1, D2, P1 and P2 time points. MEG was used in 18 subjects, on a CTF systems 274-channel whole-head MEG scanner with third-order axial gradiometres and a sampling rate of 480 Hz. Electroencephalogram (EEG) was used in two subjects (patients 8 and 19) due to MEG artefacts. EEG data were acquired with a high-density, 128-channel Bio-Semi headcap system, with a sampling rate of 512 Hz.

### MEG/EEG experimental paradigm and stimuli

Spoken auditory stimuli were presented binaurally in a passive oddball paradigm, with the standard stimulus ‘Bart’, an acoustic deviant, and two phonemic deviants ‘burt’ and ‘beat’. The deviant stimuli were created by varying the frequencies of the standard’s first and second formants, as described by Teki and colleagues.^19^ At each time point, four stimuli blocks were presented, each containing 120 presentations of the standard and 30 presentations of each deviant in a pseudo-randomised order. Stimulus onset asynchrony was 1080 ms. Stimulus amplitude was initially set at 60 dB/sound pressure level and adjusted prior to scanning to a comfortable level.

The participants were instructed to attend to an incidental visual detection task while ignoring the auditory stimuli. Pictures of outdoor scenes were presented for 60 s, followed by a circle (92% of trials) or square (8% of trials) for 1.5 s. The participants responded by button press to the circle and withheld their response for the square. Average response accuracy was 87%, with no significant differences in the number of errors (false-positives or false-negatives) as an effect of time (main effect of chronological order of testing sessions) or due to drug administration (main effect of drug vs placebo).

### MEG/EEG preprocessing

Preprocessing of MEG data in SPM8^31^ included the following: high-pass filtering (1 Hz), eye-blink artefact removal using multiple source eye correction,^32^ epoching (−100 to 500 ms peristimulus time), baseline correction (−100 to 0 ms prestimulus time window), low-pass filtering (30 Hz), merging the four blocks of each dataset, robust averaging and low-pass filtering again. Preprocessing of EEG data in SPM12^31 33^ included the following: high-pass filtering (1 Hz), epoching (−100 to 500 ms peristimulus time), merging the four runs of each dataset, identification of blinks from vertical electro-oculography data, correction of blink artefacts using single value decomposition and signal-space projection, robust averaging and low-pass filtering (30 Hz). (Note: Low-pass filtering at 30 Hz would have removed any time-locked effects in the gamma range from our analyses.)

Source localisation using the variational-Bayesian equivalent current dipoles^34^ is described in the online s upplementary material. In brief, this compared different source models, containing five possible sources identified from previous auditory MMN analyses (left and right HG, left and right STG and right IFG) in different combinations. The winning model, containing left and right HG and STG only (p=0.88), was used as the spatial model for the DCM analysis. All patients had at least 10% intact cortex within each anatomical region of interest. All fitted sources fell within intact cortex, not lesion (see online s upplementary material for detail).

### Dynamic causal modelling

DCM was conducted on preprocessed evoked responses to standard and deviant stimuli at each time point separately (D1, D2, P1 and P2). Evoked responses from 1 to 400 ms peristimulus time were tapered with a Hanning window. The DCM spatial model used Equivalent Current Dipole (ECD) sources from bilateral HG and STG dipole locations (see online supplementary material). The neural model included exogenous auditory stimulation to the left and right HG nodes at 60 ms; endogenous connectivity evoked by standard stimuli in two forward, two backward and four lateral connections between HG and STG sources (diagonal connections were not modelled because although heterotopic connections are present in auditory cortex they are sparse)^35^; and three modulatory effects evoked by each deviant stimulus compared with the standard stimulus. A large model space of 2^8 (256) models was estimated, each with modulatory effects modelled in a different combination of the eight independent inter-regional connections. Modulatory effects on self-connections, modelling a region’s sensitivity to inputs,^36^ were present in all models.

### Bayesian model averaging

For each connection, Bayesian Model Averaging (BMA) with random effects^37^ was used at the subject level to average the modulation caused by each deviant versus the standard at each time point (D1, D2, P1 and P2). The difference in modulation by phonemic versus acoustic deviants (phonemic sensitivity) was calculated by averaging the modulatory effects for the two phonemic deviants and subtracting the modulatory effect for the acoustic deviant.

The phonemic sensitivity values were entered into a repeated-measures analysis of variance (ANOVA) for each connection. The ANOVAs had within-subjects variables time (before/after phonological training) and drug (on drug/placebo), and between-subjects variables severity (moderate/severe groups) and cross-over order (drug/placebo first).

## Results

### Baseline language performance and structural MRI

**Figure 3:**
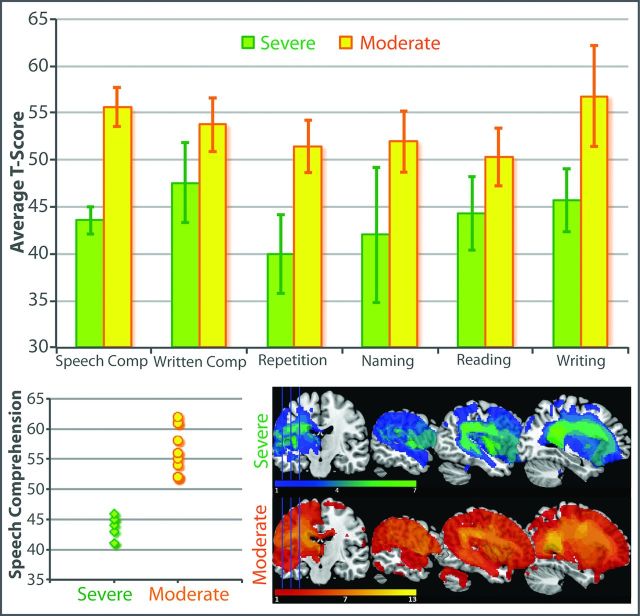
Baseline Comprehensive Aphasia Test data and structural brain imaging. Top: average T-scores for severe (n=7) and moderate (n=13) patients. Bottom left: speech comprehension T-scores, showing division of severe and moderate subgroups. Bottom right: lesion overlay maps for severe and moderate patients.

Exploratory analysis of the baseline CAT speech comprehension scores using a two-step cluster analysis identified a division between participants with moderate (n=13) and severe (n=7) impairments (figure 3). Independent samples t-tests confirmed that moderate and severe subgroups differed in speech comprehension ability (t(18)=7.77, p<0.001), as well as in written comprehension, repetition, naming, reading and writing abilities (all p<0.05). The subgroups did not differ significantly in age (p=0.35) or time since stroke (p=0.43). Lesion overlay maps (figure 3) showed the subgroups shared broadly similar lesion distributions, centring on the left perisylvian cortex and superior longitudinal fasciculus. The lesion volume was not significantly different between groups (p=0.94).

### Phonological training dose

Out of 40 completed training blocks (2 blocks × 20 participants), 28 self-report diaries were fully completed (70%). Of those 28 diaries, the average training dose was 36 hours 38 min per block (SD=16 hours 8 min; range: 6 hours 20 min to 65 hours 5 min; median=36 hours 28 min). Fifteen of the 28 diaries were from blocks 1 and 13 were from Block 2. The mean training dose for block 1 and block 2 did not differ significantly (block 1: mean=38 hours 58 min; block 2: mean=36 hours 26 min; *t*(26)=0.37, p=0.71).

### Compliance and tolerability of cholinergic drug therapy

Patients were required to return their medication bottles after completing each block. Eighty-eight per cent of medication bottles were returned. In these bottles, only 2.5% of unused tablets remained.

The participants completed an adverse effects report form at each time point (see online supplementary table). Three participants had incomplete report forms at one time point; hence, reports were analysed for 17 participants only. We compared the total number of adverse events when the participants were on drug (D1 or D2) or placebo (P1 or P2). The participants reported 32 adverse events when on drug and 28 on placebo. A non-parametric sign test showed no significant difference in the frequency of adverse events on drug versus placebo (p=0.79).

The most frequent adverse events were insomnia, headaches, dizziness and muscle cramps. For each type of event, the frequency of occurrence on drug or placebo was compared using the McNemar tests with binomial distribution. No significant differences were observed for any type of event (all p>0.2).

### Therapy effects on behavioural outcome measures: primary outcomes

**Figure 4:**
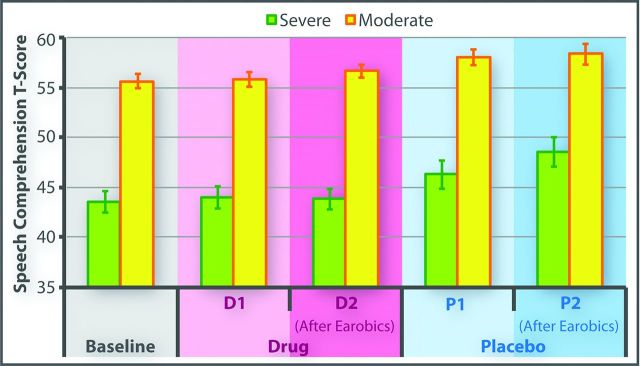
Effects of Earobics and donepezil on speech comprehension in severe and moderate patient subgroups.

The effects of phonological training and donepezil on CAT speech comprehension scores were assessed using a mixed effects ANOVA with two within-subjects variables: (1) time (before/after phonological training) and (2) drug (on drug/on placebo). Two between-subjects variables were also included: (1) aphasia severity (moderate/severe) and (2) cross-over group (drug first/placebo first). The results (figure 4) demonstrated that phonological training significantly improved speech comprehension, indicated by a significant main effect of time (F(1, 16)=6.56, p<0.05). Conversely, there was a significant main effect of drug, with (unexpectedly) lower scores on drug than placebo (F(1, 16)=11.60, p<0.005). There was no interaction between phonological training and donepezil: the effects were independent and in opposite directions. Time by severity and drug by severity interactions were significant (F(1, 16)=6.6, p<0.05 and F(1, 16)=4.9, p<0.05, respectively): both therapy effects were larger in the severe patient group.

### Therapy effects on behavioural outcome measures: secondary outcomes

Among the other CAT measures (written comprehension, speech repetition, naming, reading and writing), the only notable effect was a significant time by severity interaction for written comprehension (F(1, 16)=8.56, p<0.05), driven by larger beneficial effects of phonological training in severe patients compared with moderate patients. There was a non-significant trend towards better naming on drug than placebo (F(1, 16)=3.64, p=0.075), consistent with prior observations by Berthier and colleagues.^17 18^

There were no significant changes in the SART or ASHA FACS scores. Repeated-measures ANOVAs demonstrated no significant effects of time, drug, severity or cross-over group.

### Therapy effects on effective connectivity

MMN responses to acoustic and phonemic deviants are shown in the online s upplementary figure 1.

**Figure 5:**
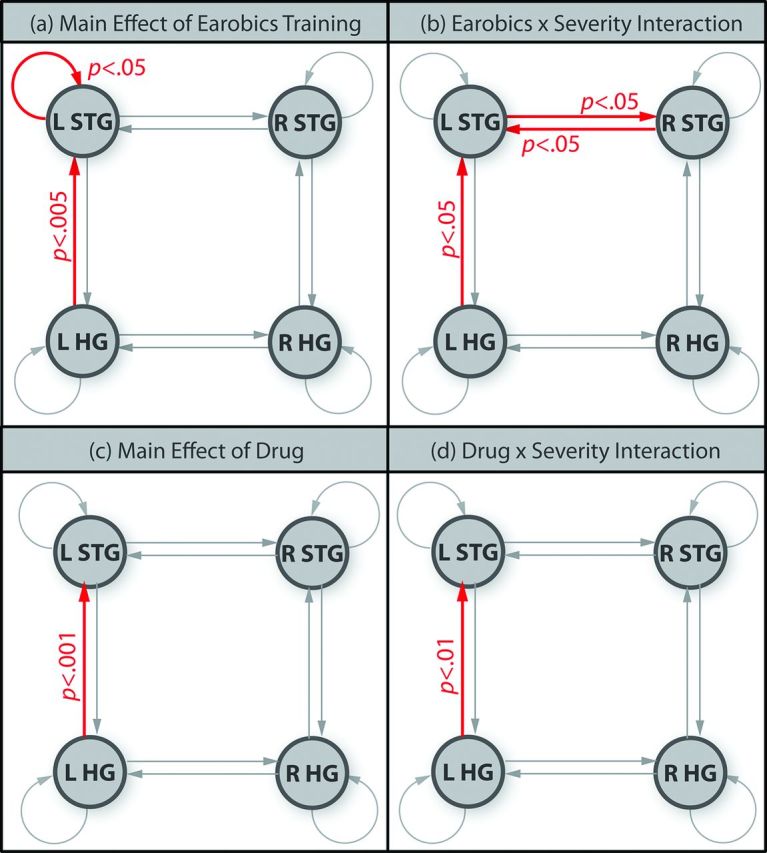
Significant changes in phonemic sensitivity between time points. (a) Red connections showed significantly stronger phonemic sensitivity after Earobics training (main effect of Earobics); (b) red connections showed significantly stronger training effects on phonemic sensitivity in the severe patients than the moderate patients (Earobics by severity interaction); (c) main effect of drug and (d) drug by severity interaction.

Modulation of connection strength by phonemic versus acoustic deviants (phonemic sensitivity) changed as a result of phonological training (figure 5). The main effects of phonological training were significant in two connections (figure 5A): (1) the left STG self-connection (F(1, 16)=5.30, p<0.05) and (2) the left HG to left STG connection (F(1, 16)=11.3, p<0.005). Three connections showed time by severity interactions driven by stronger training effects in the severe group (figure 5B): (1￼) the left HG to left STG forward connection (F(1, 16)=8.30, p<0.05) (2) the left STG to right STG lateral connection (F(1, 16)=6.65, p<0.05) and (3) the right STG to left STG lateral connection (F(1, 16)=8.64, p<0.01).

Only one connection, the left HG to STG, showed a main effect of drug (F(1, 16)=20.70, p<0.001) due to stronger phonemic sensitivity on drug versus placebo (figure 5C). A drug by severity interaction (figure 5D) showed that this effect was larger for severe than moderate patients (F(1, 16)=9.79, p<0.01).

## Discussion

We hypothesised that phonological training would improve speech comprehension and that donepezil would facilitate this effect by enhancing the neuroplastic response to training. Only the first hypothesis was supported: patients showed significantly better speech comprehension after phonological training, but worse comprehension on drug than placebo. Both effects were stronger in more severely impaired patients: the severe subgroup responded better to training and worse to drug than the moderate subgroup. The effect of phonological training on comprehension was significant but clinically small: the average CAT speech comprehension score during the placebo block increased from 52.0 to 53.2 after 5 weeks of training. The average dose was 36 hours per block. Lower doses of phonological therapy in previous studies (6–12 hours) may explain why significant effects have not been consistently observed.^10 11^ Although the effect of phonological training was small, it is of clinical importance as therapeutic interventions in patients with aphasia with severe impairments of speech perception have been largely written off, both in textbooks (‘(global aphasia is) sometimes known as irreversible aphasia syndrome’^38^) and in systematic reviews (‘No evidence of benefit’^39^).

The behavioural effect of phonological training was generalised in two ways: first, training with Earobics stimuli generalised to different stimuli used in CAT speech comprehension tests and second, written comprehension improved, at least for severe patients. This latter result suggests that either auditory training acted on amodal, higher level semantic representations or that strengthening *auditory* phonological representations also facilitates grapheme-to-phoneme translation required for written comprehension. A third option, that training improved non-linguistic, domain-general cognitive abilities, is less likely as there were no improvements on sustained attention.

The surprising result that speech comprehension was worse on donepezil than placebo contrasts with improved Western Aphasia Battery Aphasia Quotients^40^ was reported by Berthier and colleagues.^17 18^ Berthier only observed task-specific improvements for picture naming. We saw a trend towards better naming on drug than placebo, perhaps suggesting that donepezil is better suited to treating speech production than comprehension. Husain and Mehta^41^ observe that cognitive-enhancement drugs can have opposing effects on different tasks as an inverted U-shaped relationship applies between neuropharmacological enhancement and performance. Hence, if acetylcholine stimulation was already high in auditory cortex, increasing it further with donepezil could have impaired performance.

The effective connectivity (DCM) analysis examined the interactions between auditory areas. We predicted that improved speech comprehension would result from a tuning of phonological representations in auditory cortex. In DCM such tuning is expressed as a region’s self-connection, which acts as a gain mechanism^36^: that is, a post-therapy increase in a self-connection’s strength would mean that the region had become more sensitive to phonological contrasts. The results confirmed our hypothesis: improved speech comprehension after phonological training was associated with stronger modulation of the left STG’s self-connection by phonological but not acoustic deviants.

Phonological training also strengthened phonological sensitivity of the forward connection from left HG to STG, suggesting that updating of higher order representations of speech sounds in the left STG was driven by stronger feedforward prediction errors. The observation that phonological modulation of this connection was also affected by drug (which did not result in a behavioural improvement) suggests that strengthening this forward connection alone is insufficient to improve speech comprehension.

Our findings were not confined to the left hemisphere. In severe patients, training led to stronger phonemic sensitivity in the STG interhemispheric connections. This implies that after severe left temporal damage, the left STG requires more support from its right hemisphere homologue to perform phonological discrimination. The baseline data in these patients treated as a single group^19^ showed that modulation strength of the left to right STG connection correlated negatively with phonemic discrimination ability; that is, there was a decoupling of these two regions in patients with better speech perception. It is not clear whether this decoupling represented (1) the best the damaged system could currently manage, with the left and right STGs maintaining their specialised functions but no longer working in tandem or (2) a maladaptive response to left-hemisphere damage that may serve to limit further recovery, as, presumably, auditory representations in the right hemisphere do not share the linguistic specialisation of the left hemisphere. The longitudinal data presented here suggest a more complex interpretation because severity effects indicated (1) moderately affected patients do not have impaired interhemispheric connectivity and therapy effects are seen within their dominant temporal lobe and (2) in more severely affected patients interhemispheric processing of phonemic stimuli is abnormal, but can still be improved by auditory training.

In conclusion, our results demonstrate that phonological training worked at a behavioural level, but the underlying neuroanatomical mechanism varied with aphasia severity. The left STG mediated therapy effects in both patient groups: in the moderate group, this occurred independently from the influence of right temporal regions, whereas in the severe group interhemispheric interactions were also involved in promoting recovery of speech perception.
