# Supplementary material for: Auditory training changes temporal lobe connectivity in ‘Wernicke’s aphasia’: a randomised trial
Source: J Neurol Neurosurg Psychiatry. 2017 Mar 4;88(7):586–94. doi: 10.1136/jnnp-2016-314621 (PMC5659142; doi:10.1136/jnnp-2016-314621)
Supplement: Supplementary file [file jnnp-2016-314621supp001.pdf]

Title: Auditory therapy re-tunes linguistic representations in left superior temporal gyri of Wernicke's aphasia patients: A randomized trial

Running Title: Auditory therapy for Wernicke's aphasia

SUPPLEMENTARY MATERIAL

Supplementary Table

Adverse events listed in the report form and frequency of occurrence in Drug and Placebo blocks.

**Adverse event**	**Frequency**	
	**On Drug (D1 or D2)**	**On Placebo (P1 or P2)**
Insomnia	5	2
Headache	4	3
Dizziness	4	3
Muscle cramps	4	3
Nausea	3	1
Diarrhoea	2	2
Rash	2	1
Pruritus	2	0
Gastric or duodenal ulcers / gastric-intestinal haemorrhage	1	0
Vomiting	1	2
Symptomatic bradycardia	0	0
Seizures (unless already known to have coexistent epilepsy)	0	2
Syncope	0	1
Hepatitis	0	0
Anorexia	0	1
Psychiatric disturbances, e.g. hallucinations / agitation	0	2
Urinary incontinence	0	1
Other	4	4
**Total**	**32**	**28**

Mismatch Negativity Responses in Sensor Space

The Mismatch Negativity (MMN) responses in left and right temporal lobe sensors at each time-point were visualised using the following procedure. The input data were the preprocessed M/EEG timeseries from each time-point at the individual subject level. Sensor-level difference waves were created by subtracting the standard from each deviant at each sensor. The absolute value of these sensor-level difference waves were averaged across all left/right temporal sensors. The plots were normalised to account for differences in signal amplitude between subjects and across modalities: each subject's plots were normalised relative to that subject's maximum signal amplitude of the average power plot across *all* sensors. The normalized timeseries for the two phonemic deviants were averaged together. These normalised timeseries were averaged across the group to produce MMN power plots for phonemic and acoustic deviants in left and right temporal sensors as shown in Supplementary Figure 1.

The MMN responses peaked between 100 and 200ms peri-stimulus time. In alignment with results from Teki and colleagues (*20*), the acoustic deviant elicited weaker and later MMN responses than the phonemic deviants. MMN responses had stronger amplitudes in left temporal than right temporal sensors.

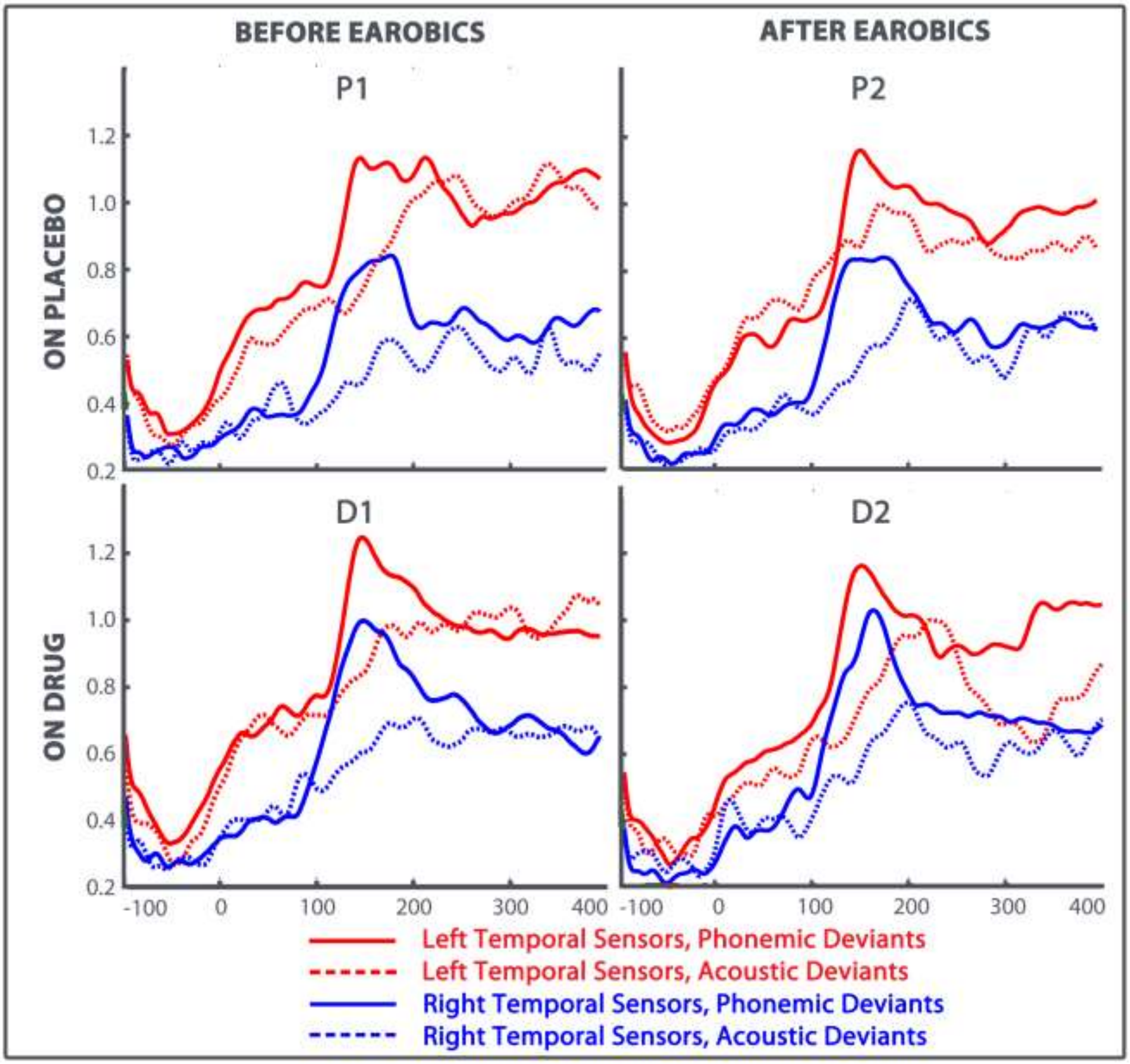

Supplementary Figure 1

Group averaged mismatch negativity (MMN) responses in sensor space. Solid lines show the MMN response for phonemic deviants versus the standard stimulus. Dashed lines show the MMN response for acoustic deviants versus the standard stimulus. Red=left temporal M/EEG sensors; Blue=right temporal sensors.

Source Localization

Dipole locations underlying the MMN response were identified using Variational Bayesian Equivalent Current Dipoles (VB-ECD)[1-2]. This approach allows subject-specific source localisation and Bayesian Model Comparison of competing spatial models. VB-ECD was performed on each subject's data at each time-point separately. The response to all three deviants were averaged to avoid biasing source locations in favour of one deviant over the others. The MMN response was defined as the first peak after the M100 auditory response; this was identified for each dataset in a semi-supervised manner.

Source location priors were identified from previous studies of MMN generators[2-4]. Four spatial models were compared:

- Model 1: left Heschl's gyrus (left HG, with prior MNI co-ordinates -42 -22 -7), right HG (right HG, 46 -14 8) and left superior temporal gyrus (left STG, -61 -32 8)
- Model 2: Left HG, right HG and right STG (right STG, 59 -25 8)
- Model 3: Left HG, right HG, left STG and right STG
- Model 4: Left HG, right HG, left STG, right STG and right inferior frontal gyrus (46 20 6).

Each spatial model was fitted in a VB-ECD analysis with 1000 iterations. Each iteration randomly selected start locations for each dipole from a Gaussian distribution with standard deviation of 6mm around the prior location. The winning solution for each analysis was the iteration with the highest model evidence conforming to the following rules: a) all sources within intact brain tissue; b) all pairs of sources separated by >1cm; and c) all sources within the regions' anatomical boundaries defined by the Harvard-Oxford cortical atlas.

The four spatial models were compared at the group level using fixed-effects Bayesian Model Comparison[5]. The exceedance probabilities of the models were P=0.1173, 0.0002, 0.8825 and 0 respectively, indicating that Model 3, with bilateral HG and STG sources, was the most probable fit to the observed data. Hence, each subject's source locations from Model 3 were used in the Dynamic Causal Modelling analysis.

REFERENCES

1. Kiebel SH, Daunizeau J, Phillips C, et al. Variational Bayesian inversion of the equivalent current dipole model in EEG/MEG. *NeuroImage* 2008;39:728-741.
2. Teki S, Barnes GR, Penny WD, et al. The right hemisphere supports but does not replace left hemisphere auditory function in patients with persisting aphasia. *Brain* 2013;136(6):1901-1912.
3. Garrido MI, Kilner JM, Kiebel SJ, et al. Dynamic causal modelling of evoked potentials: A reproducibility study. *NeuroImage* 2007;36:571-580.
4. Opitz B, Rinne T, Mecklinger A, et al. Differential contribution of frontal and temporal cortices to auditory change detection: fMRI and ERP results. *NeuroImage* 2002;15:167-174.
5. Penny WD, Stephan KE, Mechelli A, Friston KJ. Comparing dynamic causal models. NeuroImage 2004;22(3):1157-1172.
